# AMPA Receptor Phosphorylation and Synaptic Colocalization on Motor Neurons Drive Maladaptive Plasticity below Complete Spinal Cord Injury

**DOI:** 10.1523/ENEURO.0091-15.2015

**Published:** 2015-11-16

**Authors:** J. Russell Huie, Ellen D. Stuck, Kuan H. Lee, Karen-Amanda Irvine, Michael S. Beattie, Jacqueline C. Bresnahan, James W. Grau, Adam R. Ferguson

**Affiliations:** 1 Department of Neurological Surgery, Brain and Spinal Injury Center, University of California San Francisco, San Francisco, California 94110; 2 Department of Neurobiology, University of Pittsburgh, Pittsburgh, Pennsylvania 15261; 3 San Francisco Veterans Affairs Medical Center, San Francisco, California 94121; 4 Department of Psychology, Texas A&M University, College Station, Texas 77841

**Keywords:** AMPA receptor, motor neuron, nociception, plasticity, spinal cord injury, spinal learning

## Abstract

Clinical spinal cord injury (SCI) is accompanied by comorbid peripheral injury in 47% of patients. Human and animal modeling data have shown that painful peripheral injuries undermine long-term recovery of locomotion through unknown mechanisms. Peripheral nociceptive stimuli induce maladaptive synaptic plasticity in dorsal horn sensory systems through AMPA receptor (AMPAR) phosphorylation and trafficking to synapses. Here we test whether ventral horn motor neurons in rats demonstrate similar experience-dependent maladaptive plasticity below a complete SCI *in vivo*. Quantitative biochemistry demonstrated that intermittent nociceptive stimulation (INS) rapidly and selectively increases AMPAR subunit GluA1 serine 831 phosphorylation and localization to synapses in the injured spinal cord, while reducing synaptic GluA2. These changes predict motor dysfunction in the absence of cell death signaling, suggesting an opportunity for therapeutic reversal. Automated confocal time-course analysis of lumbar ventral horn motor neurons confirmed a time-dependent increase in synaptic GluA1 with concurrent decrease in synaptic GluA2. Optical fractionation of neuronal plasma membranes revealed GluA2 removal from extrasynaptic sites on motor neurons early after INS followed by removal from synapses 2 h later. As GluA2-lacking AMPARs are canonical calcium-permeable AMPARs (CP-AMPARs), their stimulus- and time-dependent insertion provides a therapeutic target for limiting calcium-dependent dynamic maladaptive plasticity after SCI. Confirming this, a selective CP-AMPAR antagonist protected against INS-induced maladaptive spinal plasticity, restoring adaptive motor responses on a sensorimotor spinal training task. These findings highlight the critical involvement of AMPARs in experience-dependent spinal cord plasticity after injury and provide a pharmacologically targetable synaptic mechanism by which early postinjury experience shapes motor plasticity.

## Significance Statement

Recent findings have demonstrated that painful stimuli below a spinal cord injury can affect future locomotor training and recovery in spinal cord injured patients (Bouffard et al., 2014), as well as in animal models (Ferguson et al., 2008a; Ferguson et al., 2012a; Ferguson et al., 2012b). However, the cellular and molecular mechanisms for this experience-dependent modulation of spinal cord plasticity are poorly understood. This work uncovers a novel synaptic mechanism by which peripheral nociceptive (painful) input following a spinal cord injury can undermine future adaptive spinal cord plasticity, providing a novel target for improving recovery after spinal cord injury, and mitigating aberrant forms of spinal neuroplasticity.

## Introduction

In the human spinal cord injury (SCI) population there is a high incidence of concomitant peripheral injury, including lacerations, abrasions, and fractured limbs (Saboe et al., 1991; Sekhon and Fehlings, 2001; Wang et al., 2001; Hasler et al., 2011). Although these injuries may be considered minor in comparison to the SCI, the nociceptive input to the spinal cord produced by these peripheral injuries may have a lasting effect on recovery of sensory and motor function. A large body of preclinical work has demonstrated that peripheral nociceptive input produces lasting alterations in dorsal horn neurons through cellular mechanisms similar to those underlying hippocampal learning and memory (Sandkühler and Liu, 1998; Ikeda, 2006). These spinal cord “pain memory” signatures have been suggested to underlie pain disorders, such as hyperalgesia and neuropathic pain (Ji et al., 2003; Sandkühler, 2009; Ruscheweyh et al., 2011; Drdla-Schutting et al., 2012).

Peripheral nociceptive input affects not only dorsal pain pathways, but also spinal motor systems. Peripheral intermittent nociceptive stimulation (INS) below a SCI produces nociceptive hyper-reflexia, acute deficits in adaptive spinal motor learning, and lasting (>6 weeks) impairment in recovery of locomotor function (Grau et al., 1998, 2004,2012; Garraway et al., 2011, 2014; Huie et al., 2012b). Such findings suggest that the injured spinal cord is highly vulnerable to peripheral sensory input, with a heightened capacity for the spinal motor systems to encode maladaptive forms of synaptic plasticity. The clinical relevance of peripherally-induced spinal cord maladaptive plasticity was highlighted by a recent clinical study demonstrating that brief peripheral nociceptive input undermines the ability of human SCI patients to retain locomotor learning tasks (Bouffard et al., 2014).

Although a growing body of work has sought to determine the neural mechanisms underlying maladaptive plasticity in pain pathways, it is unknown whether nociception-induced alterations in motor circuits reflect these same processes. One key aspect of spinal cord pain plasticity is the postsynaptic alteration of fast-excitatory glutamate AMPA receptors (AMPARs; Hartmann et al., 2004; Galan et al., 2004). Recent work has also highlighted the specific role of AMPAR subunit phosphorylation site serine 831 in encoding activity-dependent spinal cord pain memory traces in dorsal spinal neurons following peripheral stimulation (Drdla-Schutting et al., 2012).

It has been recently shown that SCI specifically increases postsynaptic localization of GluA2-lacking, calcium-permeable AMPA receptors (CP-AMPARs) on motor neurons (Ferguson et al., 2008b). In addition, AMPAR activity impacts spinal cord motor training, suggesting potential for dynamic modulation of AMPARs in spinal cord motor systems (Hoy et al., 2013). The synaptic insertion and activation of CP-AMPARs are crucial to the induction of synaptic plasticity and homeostatic scaling in CNS neurons *in vitro* (Man, 2011; Garcia-Bereguiain et al., 2013). CP-AMPARs are normally highly regulated, and are trafficked out of synaptic sites; however, under pathophysiologic conditions, CP-AMPAR activity may be dysregulated and sustained, overdriving excitation and ultimately inducing cell death (Kuner et al., 2005; von Lewinski and Keller, 2005; Liu et al., 2006; Ferguson et al., 2008b; Oh et al., 2012; Spaethling et al., 2012; Dias et al., 2013).

Given these prior findings, we hypothesized that nociceptive input induces maladaptive spinal cord plasticity by overdriving CP-AMPARs in motor neurons in the injured spinal cord. We applied a well established peripheral nociceptive stimulation procedure to induce maladaptive motor plasticity, and tested for AMPAR and downstream signaling changes by quantitative biochemistry, robotic confocal image analysis, and behavioral assessments at acute time-points following complete SCI. The results uncovered a time dependent overdrive of CP-AMPARs that was independent of cell death and pharmacologically targetable to reset spinal cord plasticity below SCI.

## Materials and Methods

### Animals

Male Sprague-Dawley rats (Harlan), aged 100–120 d were housed individually and had *ad libitum* access to food and water (*N* = 64). All procedures were conducted in accordance with the National Institute of Health *Guide for the Care and Use of Laboratory Animals*, and all efforts were made to minimize suffering and limit the number of animals used. All protocols were approved by the University Laboratory Animal Care Committee at Texas A&M University.

### Experimental methodology

In all experiments, experimenters were kept blind to treatment conditions throughout the entire study in accordance with recent quality standards for preclinical neurological research (Landis et al., 2012). Western blot loading order was determined *a priori* by a third-party coder who insured that a representative sample from each condition was included on each gel in a randomized block design. For each experiment, the number of subjects per condition was kept consistent across groups to insure proper counterbalancing could be achieved across independent Western runs. All representative Western images presented in the figures represent lanes from the same gel. Because of our randomized counterbalancing scheme, occasionally the critical comparisons of interest were not available on adjacent lanes (but do come from the same gel). The entire set of randomized Western blot images are available upon request. Confocal image acquisition and subsequent data analyses were also performed by experimenters blind to treatment condition.

### Spinal transection surgery

All animal subjects received a complete transection of the spinal cord immediately rostral to the second thoracic vertebra (T2). Animals were fully anesthetized with isoflurane gas (5%). Fur over the thoracic vertebra was shaved, and a 3 cm incision was made over T2. The tissue immediately rostral to T2 was cleared away with rongeurs, and the underlying spinal cord was exposed. An electrical heat cautery device was used to transect the spinal cord and the resulting cavity was filled with gelfoam (Harvard Apparatus). The incision was then closed using Michel clips (Fine Science Tools). Animals received a 2.5 ml intraperitoneal injection of 0.9% saline immediately following surgery, and twice daily for subsequent days to ensure proper hydration. Bladders were expressed twice daily. Given the nociceptive nature of this study, no analgesics were given following complete spinal transection.

### Intermittent nociceptive stimulation

Twenty-four hours after complete spinal transection surgery, animals were placed in black Plexiglas tubes, 22 cm in length and 6.8 cm in diameter. The tail was allowed to hang freely from the end of the tube, and an electrode coated in ECG gel was fixed to the tail ∼6 cm from the base of the tail using orthaletic tape. Constant-current 1.5 mA, AC stimulation was delivered to the electrode using a 660 V transformer. Stimulation delivery was controlled by computer program, with each pulse 80 ms in duration delivered intermittently over 6 min, on a variable interstimulus interval (range 0.2–3.8 s; mean 2 s). This schedule resulted in a total of ∼180 stimulation presentations. Each animal received either a single 6 min stimulation session, or an equivalent period of unstimulated restraint with the electrodes attached.

### Western blot

Animals were deeply anesthetized with pentobarbital (100 mg/kg, i.p.), decapitated, and spinal cords were harvested by rapid fluid expulsion with ice-cold phosphate-buffered saline (PBS) at 20 min, 2 h, or 24 h following nociceptive stimulation. Spinal cords were immediately flash-frozen in liquid nitrogen, and then transferred to −80°C. The entire surgical procedure was timed to ensure time from decapitation to snap freeze was <5 min.

**Figure 1.**
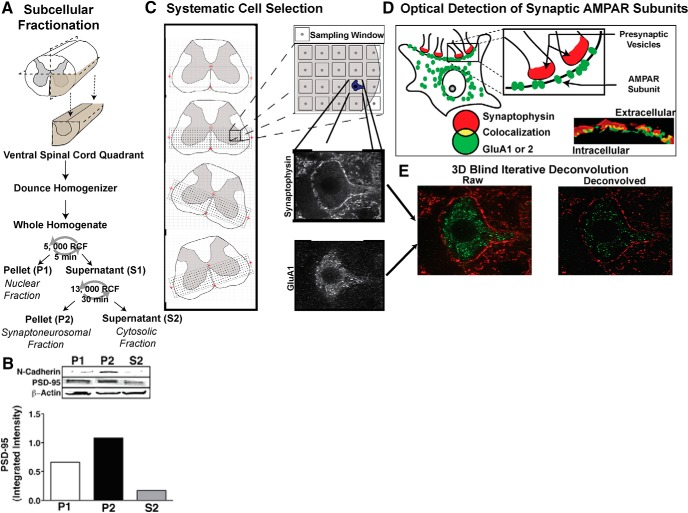
Subcellular fractionation of ventral lumbar spinal cord. *A*, Ventral quadrant of lumbar spinal cord tissue was dissected and homogenized using a Dounce homogenizer. The whole homogenate was then centrifuged for 5 min at 5000 rcf, and the supernatant (S1) was removed. This S1 fraction was then centrifuged for 30 min at 13,000 rcf. The pellet from this fraction (P2) was then used for all subsequent Western blots. ***B***, Plasma membrane enrichment in the P2 fraction was confirmed with *N*-cadherin expression, and modest synaptic enrichment (synaptoneurosome) was characterized by PSD-95, with beta-actin serving as loading control. ***C***, Tissue-oriented coordinate grid placement for systematic cell selection. Large ventral horn neurons were systematically selected from spinal cord slices in L4–L5 region. A microscopist blind to experimental condition entered the stage coordinate location of four anatomical landmarks (central canal, anterior artery, left edge of tissue, right edge of tissue) into an Excel spreadsheet, and a custom VisualBasic macro generated a list of microscope stage coordinates that were then input into MicroManager and ImageJ software that controlled microscope stage movement. This system ensured that the orientation of the coordinate grid would always be relative to the specific orientation of the tissue. Each coordinate signified the center of an 80 × 80μm sampling window, with each coordinate spaced 100 μm apart. The blind microscopist cycled through these sampling windows at 63×. When a large neuron (cell body >40 μm) was encountered within a sampling window, the cell was centered in the frame and a stack of images was taken through the *z*-plane, with separate images taken through a 650 nm filter (for synaptophysin) and a 490 nm filter (for GluA1 or GluA2) at each level. ***D***, Optical detection of synaptic AMPAR subunits. Yellow pixels produced by the overlapping of the presynaptic marker synaptophysin (red) and postsynaptic AMPAR subunit (green) puncta indicated colocalization and were quantified to determine the amount of synaptic AMPAR subunit expression. ***E***, All image stacks were combined and deconvolved to correct for the diffusion of light using AutoQuant software.

Fresh-frozen spinal cords were subsequently rapidly thawed on a chilled petri dish at 4°C, and a 1 cm section of the lumbar enlargement was dissected. This section was then split once along the midline, followed by a cut to separate the dorsal and ventral quadrants (Fig. 1*A*). A single quadrant containing the ventral horn was then placed in a Dounce homogenizer filled with 200 μm homogenization buffer (10 mm Tris, 30 mm sucrose, pH 7.5) containing phosphatase and protease inhibitors (Roche). Following tissue disruption, the whole homogenate was placed in an Eppendorf tube, and spun at 5000 relative centrifugal force (rcf) for 5 min in a minicentrifuge at 4°C. This centrifugation procedure produced a supernatant (S1) and a nuclear pellet (P1). The S1 layer was removed and centrifuged a second time at 13,000 rcf for 30 min at 4°C, producing a modestly synaptoneurosomal-enriched pellet fraction (P2) that was subsequently used in Western blots (Fig. 1*B*; Galan et al., 2004; Ferguson et al., 2008b; Stück et al., 2012). Total protein concentration was quantified using the Pierce BCA protein assay method. Each sample was then diluted 1:2 in room temperature Laemmli sample buffer, and 20 μg of total protein per sample was loaded into separate lanes on a precast 10–20% electrophoresis gel (Tris-HCl polyacrylamide, BioRad). Samples were counterbalanced across the gel by treatment condition, and the experimenter was kept blind to condition. A kaleidoscope ladder was loaded on each gel to confirm molecular weight. The gel was electrophoresed for 1 h at 100 V in SDS buffer (25 mm Tris, 192 mm glycine, 0.1% SDS, pH 8.3; BioRad). Protein was transferred to a nitrocellulose membrane in cold transfer buffer (25 mm Tris, 192 mm glycine, 20% methanol, pH 8.3). The membrane was blocked for 1 h in Odyssey Blocking Buffer (LiCor) containing 0.1% Tween 20, followed by an overnight incubation in primary antibody solution at 4°C. Primary antibody solution consisted of rabbit polyclonal anti-phosphorylated serine 831 (1:200; Millipore), in Odyssey blocking buffer plus 0.05% Tween 20. Following incubation, the membrane was washed 4 × 5 min with Tris-buffered saline containing 0.1% Tween 20 (TTBS) and incubated in fluorescent-labeled secondary antibody (1:30K LiCor IRdye 680 goat anti-rabbit in Odyssey blocking buffer plus 0.2% Tween 20) for 1 h in the dark. Subsequent to 4 × 5 min washes in TTBS, followed by a 5 min wash in TBS. The membrane was immediately scanned on the LiCor Odyssey Infrared Imaging System with a 680 nm laser to reveal protein bands. The membrane was then re-blocked and reincubated with mouse anti-GluA1 primary antibody (1:200, Millipore) in Odyssey blocking buffer plus 0.05% Tween 20. The membrane was then washed as before, and incubated in fluorescent-labeled secondary antibody (1:30K LiCor IRdye 800 goat anti-mouse in Odyssey blocking buffer plus 0.2% Tween 20), washed as before, and rescanned with the 800 nm laser to reveal GluA1 protein bands. This same protocol was performed for serine 845 on GluA1, GluA2, and Serine 880 on GluA2. Loading controls were performed as the last step on each multiplexed Western blot. Cell death marker assays (cFos, cJun, calpain I, and cleaved caspase, 1:250; Cell Signaling Technologies), were performed on a separate set of gels that were performed on cytosolic (S2) fractions. Otherwise, the Western blot protocols for cell death markers were identical to those used for AMPAR assays.

To ensure accurate protein quantification, we used protein dilution curves for each primary antibody to detect the linear range in which fluorescence was most highly correlated with changes in protein concentration using Odyssey Application Software v3.0. The optimal dilution and laser intensity at which protein concentration and fluorescence yielded the greatest linear correlation was established separately for each antibody and then held constant for the duration of the experiment (all *R* ^2^ > 0.98). Fluorescence for each band was quantified using Odyssey software and normalized to median pixel density of background fluorescence.

### Immunohistochemistry

Animals (*n* = 4 per group) underwent intracardial perfusion under deep (100 mg/kg) pentobarbital anesthesia, either 20 min or 2 h post-INS. Animals were perfused first with ice cold PBS, followed by 4% paraformaldehyde to fix tissue. The spinal cord was excised and postfixed overnight (<18 h) in 4% paraformaldehyde then cryoprotected in 30% sucrose for 2 d. The tissue was embedded in OCT within 10 mm blocks, flash frozen on dry ice, and sectioned into 20-μm-thick coronal slices.

The L4–L5 region of the spinal cord was identified anatomically using Luxol Fast Blue (LFB) staining. The tissue was dried for 1 h and hydrated with 1:1 95% ethanol to chloroform for 4 h. Slides were incubated in a solution of 0.1% LFB for 18 h at 56°C. The tissue was then washed in water and the LFB stain was differentiated by a 20 s 0.1% lithium carbonate wash. The tissue was washed again with water. Tissue was soaked in 95% ethanol for 2 min, 100% ethanol for 5 min, and another bath of 100% ethanol for 5 min. Slides were coverslipped with DPX mounting medium.

Four fixed-tissue sections from the L4–L5 region of the spinal cord from each subject were antibody-labeled using a high-throughput staining station (Sequenza; Thermo Scientific). Tissue was blocked and permeabilized with 5% normal goat serum and 0.3% Triton X-100 for 1 h and incubated overnight at room temperature in primary antibody solution consisting of mouse monoclonal anti-presynaptic synaptophysin (1:200; Millipore) and either rabbit monoclonal antibody against GluA1 (1:200; Upstate) or rabbit polyclonal antibody against GluA2 (1:200; Chemicon). Slides were washed 2 × 5 min with 2 ml PBS then incubated for 1 h at room temperature in a secondary solution containing 1:100 AlexaFluor 488 goat anti-rabbit and 1:100 AlexaFluor 633 goat anti-mouse secondary antibodies. Slides were washed 2 × 5 min with 2 ml PBS, and then coverslipped with Vectashield containing DAPI (4′, 6-diamidino-2-phenylindole; Vector Laboratories). AMPAR GluA1 and GluA2 subunits were assessed on adjacent slide sets from the same subjects.

### Robotic confocal microscopy sampling and deconvolution

A Nikon spinning disk confocal microscope (63× objective; NA = 1.4; 2× zoom) was used to collect confocal images of large ventral neurons. To systematically sample motor neurons, a custom Visual Basic program was used to automatically overlay a sampling grid on the ventral half of each spinal section, aligned to the central canal and ventral artery as fiduciary points. This grid contained 90 × 90 μm sampling windows that were spaced 100 μm apart both horizontally and vertically (Fig. 1*C*). Large neurons were identified based on the distinctive synaptophysin outline surrounding the plasma membrane and characterized by a cell body diameter >40 μm (Fig. 1*C*). A microscopist blind to condition used ImageJ software (v1.47, NIH) on the confocal microscope to advance through each sampling window, and gathered confocal images of neurons if they were encountered in the sampling window. If a sampling window contained multiple neurons, a single neuron was chosen at random. Control tissue was used to optimize filter and laser settings, which were then held constant throughout the experiment. Confocal *z*-stacks consisted of slices collected at 0.25 μm z-intervals. Confocal stacks were batch deblurred off-line using 3-D blind iterative deconvolution (AutoQuant; Fig. 1*E*). Based on a random subset of images an experimentally blind rater determined the number of iterations for the deconvolution algorithm to yield maximal resolution for each antibody (iteration = 4 for GluA1; iteration = 2 for GluA2). Once selected *a priori*, iteration numbers were held constant throughout the experiment. A total of 539 cells were assessed for GluA1, and 484 cells were assessed for GluA2. A total of 23,723 optical planes were analyzed, with an average number of 23 optical planes per cell.

We used automated image analysis to quantify the number of fluorescently labeled AMPAR puncta that exceeded a predetermined pixel threshold that was based on control tissue. Automated image analysis was performed using custom-designed MetaMorph (Molecular Devices) macros. One macro quantified AMPAR puncta at the level of the neuropil. This macro measured the amount of total AMPAR pixels as well as colocalized synaptophysin and AMPAR pixels in each plane of the *z*-stack. Another macro quantified fluorescently labeled AMPAR puncta on the plasma membrane of motor neurons. This macro first identified the plane in the *z*-series with the highest amount of synaptophysin and AMPAR subunit (GluA1 or GluA2) synaptic colocalization. The automated plane selection was monitored by a blinded researcher to prevent selection based on staining artifacts. A blinded researcher traced the synaptophysin labeled plasma membrane of the motor neuron on a single optical plane. The macro then generated a 2-µm-thick cutout containing the plasma membrane “optical fraction” of the cell from the single plane. From the plasma membrane containing cutout, MetaMorph quantified total AMPAR subunit pixels and colocalized AMPAR pixels with synaptophysin pixels (Fig. 1*D*).

### Intrathecal drug delivery

To inhibit CP-AMPAR activity, the specific antagonist Naspm (Sigma-Aldrich) was delivered intrathecally. A subset of animals (*n* = 12 per group) were fitted with an intrathecal cannula at the time of spinal transection. The 25 cm cannula was threaded into the subarachnoid space at the second thoracic vertebra, and slid 9 cm caudally so that the tip of the cannula rested on the dorsal surface of the L4–L5 section of the spinal cord. Immediately following nociceptive stimulation, a 10 μl Hamilton syringe was fitted onto the exposed end of the cannula, and 10 μl of either Naspm (10 mm) or saline vehicle was delivered over the 3 min. This injection was followed by a 20 μl flush with saline, and the exposed end of the cannula was then heat-sealed.

### Spinal sensorimotor task

Testing of spinal learning was conducted 24 h after complete spinal transection. Animals were loosely restrained in black Plexiglas tubes (22 cm in length × 6.8 cm in diameter). To limit the effects of trunk movement on hindlimb position while in the tube, a wire belt was wrapped around the animal’s trunk. Hindlimbs were allowed to hang freely from the tube. One hindlimb was shaved, and a wire electrode was inserted through the skin over the ankle, distal to the tibialis anterior muscle. A second electrode was inserted into the body of the tibialis anterior muscle, ∼1.7 cm from the first electrode. This second electrode was connected to a wire running to a BRS/LVE constant current shock generator (60 Hz, AC). A stainless steel contact electrode (7 cm in length, 0.46 cm in diameter) was secured to the hindpaw between the second and third digits with a piece of porous orthaletic tape. A wire extending from the contact electrode was connected to a computer-monitored digital input. Prior to testing, intensity of the stimulation was adjusted for each animal so that a 0.4 N force in flexion was elicited. Force was recorded by attaching a monofilament plastic line around the animal’s hindpaw, and running this line to a strain gauge (Fort-1000, World Precision Instruments). This strain gauge was connected to a multimeter calibrated to convert a change in voltage to force in Newtons. Brief (300 ms) shock pulses were then delivered to the anterior tibialis muscle, and the force of ankle flexion was recorded. Intensity was then adjusted until a 0.4 N force in flexion was achieved. After the intensity of stimulation was determined to elicit a 0.4 N flexion, the monofilament line was removed.

A rectangular plastic dish containing a salt solution was positioned 7.5 cm below the restraining tube. The level of salt solution was adjusted so that the contact electrode that extended from the rat’s hindpaw was submerged 4 mm in the solution. A stainless steel rod (1 mm in diameter) connected to a ground wire was then placed in the salt solution and the computer-controlled 30 min testing session was started. Whenever the contact electrode was submerged in the salt solution, a circuit was completed, the state of which was relayed to the stimulator, and stimulation was delivered to the tibialis anterior. Whenever the contact electrode was raised from the salt solution, the stimulation was terminated.

Three behavioral measures were taken during the testing session: time in solution, response number, and response duration. The computer monitoring the state of the circuit recorded the total amount of time that the contact electrode was in the salt solution for each 1 min time bin. Each time the contact electrode was lifted from the salt solution, response number was increased. Response duration was calculated from time in solution and response number according to the equation: Response Duration*_i_* = (60 s − Time in Solution*_i_*)/(Response Number + 1), where *i* is the current 1 min time bin.

### Statistical analyses

Statistical analyses were performed using SPSS Statistics (v.20, IBM). Parametric Western blot data for each experiment were pooled across three independent replications and covariance corrected by beta-actin loading control, with replication statistically controlled as a random factor. A mixed-model analysis of covariance (ANCOVA; with beta-actin loading control as covariate) was used to test for main effects and interactions between time and stimulation condition. Tests of main effects were followed by planned pairwise comparisons of control/experimental group estimated marginal means at each timepoint (least significant difference). Confocal and behavioral data were assessed by ANOVA, followed by Tukey’s *post hoc* tests of group means. Ratio data were analyzed with Student’s *t* test, followed by Mann–Whitney *U* to rule out nonparametric effects. Significance was assessed at *p* < 0.05. Power and effect sizes for analyses can be found in Table 1, and referred to by superscript letters throughout the results section.

**Table 1:** Statistical Analyses

Results	Type of Test	Effect Size (Eta Squared)	Observed Power
a GluA1 Western blot, Time × Stimulation interaction	ANCOVA	0.231	0.940
b GluA2 Western blot, Time × Stimulation interaction	ANCOVA	0.025	0.178
c GluA1: GluA2 ratio	t test	0.592	0.694
d p-S831 Western blot, Time × Stimulation interaction	ANCOVA	0.273	0.975
e p-S880 Western blot, Time × Stimulation interaction	ANCOVA	0.067	0.406
f Western blot cell death markers	ANCOVA	<0.023	<0.170
g GluA1 neuropil expression, main effects of time and stimulation	ANOVA	Effect of time: 0.066 Effect of stimulation: 0.053	Effect of time: 1.000 Effect of stimulation: 1.000
h GluA2 neuropil expression, Time × Stimulation interaction	ANOVA	0.036	0.991
i GluA1 extrasynaptic membrane expression, Time × Stimulation interaction	ANOVA	0.016	0.847
j GluA1 synaptic expression, main effects of time and stimulation	ANOVA	Effect of time: 0.111 Effect of stimulation: 0.015	Effect of time: 1.000 Effect of stimulation: 0.800
k GluA2 extrasynaptic membrane expression, main effects of time and stimulation	ANOVA	Effect of time: 0.030 Effect of stimulation: 0.125	Effect of time: 0.973 Effect of stimulation: 1.000
l GluA2 synaptic expression, Time × Stimulation interaction	ANOVA	0.035	0.988
m Response duration, sensorimotor learning task, Time × Drug interaction	ANOVA, repeated measures	0.069	0.991

## Results

### INS drives GluA1 but not GluA2 subunits into synaptoneurosomes in a time-dependent fashion

**Figure 2.**
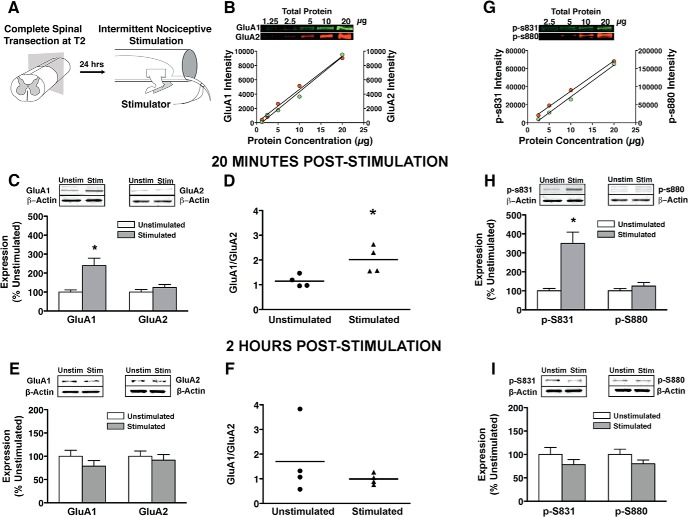
Plasma membrane GluA1 and GluA2 phosphorylation with intermittent nociceptive stimulation delivered below complete spinal cord injury. ***A***, Intermittent nociceptive stimulation. Rats with complete spinal transections received 6 min of intermittent nociceptive stimulation to the tail. Unstimulated controls received an equivalent period of restraint. ***B***, Quantitative fluorescent intensity optimized for linear detection of each target band using a 1:2 dilution curve of total protein. Laser scanning intensity for each target protein was chosen based on closest linear relationship between fluorescent intensity and total protein (all *R* ^2^ > 0.98). All subsequent analyses for each target protein were run at their respective optimal scanning intensities to ensure linearity of fluorescence (see Materials and Methods for details). ***C***, Linear quantification of GluA1 and GluA2 AMPAR subunits, 20 min poststimulation. Stimulation significantly increased GluA1 expression compared to unstimulated controls (*p < 0.05), whereas GluA2 expression was unchanged. ***D***, The ratio of GluA1 to GluA2 subunit expression was significantly increased in stimulated animals within 20 min (Mann–Whitney *U*; *p < 0.05). ***E***, ***F***, Linear quantification of GluA1 and GluA2 AMPAR subunits, 2 h poststimulation. Stimulation had no significant effect on GluA1 or GluA2 after 2 h (*p* > 0.05). ***G***, Linear intensity optimization on phosphorylated serines 831 and 880. ***H***, Linear quantification of p-S831 and p-S880 protein expression 20 min poststimulation. Stimulation significantly increased phosphorylated serine 831 expression relative to unstimulated controls (*p < 0.05), whereas phosphorylated serine 880 was unchanged. ***I***, Stimulation had no significant effect on p-S831 or pS880 after 2 h (*p* > 0.05). All bars represent mean for *n* = 4 subjects/per group (*n* = 8 for main effects, *n* =4 for interaction) with three independent Western blot runs per subject. Error bars represent standard error of the mean.

To assess the role of AMPAR activity in maladaptive spinal plasticity we applied an INS procedure (Fig. 2*A*) that induces maladaptive behavioral effects, including nociceptive hyper-reactivity, impaired spinal cord motor training, and impaired recovery of function after SCI (Crown et al., 2002b; Grau et al., 2004; Ferguson et al., 2006), and measured AMPAR changes within lumbar spinal cord synaptoneuromes by quantitative near-IR Western blotting (Fig. 2*B*). Time-series data indicated that INS increases GluA1 subunit localization to synaptic fraction by 20 min with no change in GluA2 (Fig. 2*C*). ANCOVA (with beta-actin as covariate) revealed a significant Time × Stimulation interaction for GluA1 (*F*_(1,43)_ = 13.10^a^, *p* < 0.05), but not GluA2 (*F*_(1,43)_ = 1.12^b^, *p* > 0.05). A Student’s *t* test revealed a significant increase in the GluA1/GluA2 ratio at 20 min post-INS (Fig. 2*D*; *p <* 0.05^c^). A Mann–Whitney *U* test was also used to rule out any nonparametric effects, and this test confirmed the *t* test result. These effects resolved by 2 h poststimulation (Fig. 2*E*,*F*). Pairwise comparisons at each time point revealed a significant increase in GluA1 in stimulated animals at 20 min (Fig. 2*C*; *p* < 0.05), but not at 2 h (Fig. 2*E*; *p* > 0.05). In contrast, there was no significant pairwise effect of stimulation on GluA2 at either time point (Fig. 2*C*,*E*; *p* > 0.05). Together the results suggest that INS drives GluA2-lacking AMPARs into synapses in the ventral spinal cord in a time-dependent fashion.

### Serine 831 phosphorylation of synaptic GluA1 in the injured spinal cord

**Figure 3.**
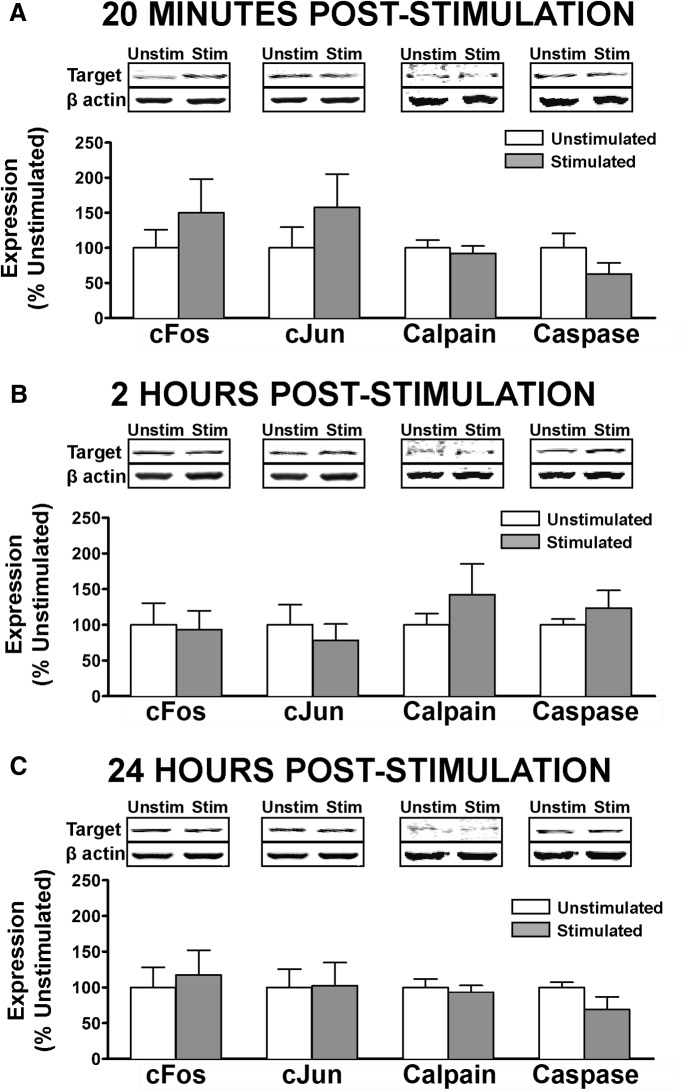
Assessment of neuronal activity and cell death markers following nociceptive stimulation. Linear Western blot quantification of cell activity and death in cytosolic fractions of ventral lumbar spinal cord, assessed at (***A***) 20 m, *(****B***) 2 h, or (***C***) 24 h after intermittent nociceptive stimulation. ANOVA showed no significant increase in broad neuronal activity marker cFos, in other more specific markers of apoptotic cell death (cJun, cleaved caspase3) or in calcium-mediated cell death (calpain I). No significant differences were observed for beta-actin loading control (*p >* 0.05). Bars represent mean for *n* = 4 subjects/factorial group (*n* =12 for INS main effect; *n* = 8 for time main effect; *n* = 4 for interaction) with three independent Western blot runs per subject. Error bars represent standard error of the mean.

Given that GluA2-lacking AMPARs are calcium permeable (Hollmann et al., 1991), the current study provide a potential mechanism for prior findings that INS engages calcium-dependent kinases, protein kinase C (PKC), and calcium-calmodulin-dependent kinase II (CaMKII; Baumbauer et al., 2007; Ferguson et al., 2008a; Huie et al., 2012a). PKC and CamKII modulate ongoing AMPAR activity by phosphorylating serine 831 (p-S831) on GluA1 and serine 880 (p-880) GluA2 (Roche et al., 1996; Matsuda et al., 1999). Quantitative phospho-Western blot of these targets revealed that INS transiently increased p-S831 on GluA1 by 20 min resolving by 2 h (Fig. 2*H*,*I*). No change was observed in p-S880 on GluA2 (Fig. 3*H*,*I*). ANCOVA (beta-actin covariate) confirmed Time × Stimulation interaction for GluA1 p-S831 (*F*_(1,43)_ = 16.10^d^, *p* < 0.05), but no effect for GluA2 p-S880 (*F*_(1,43)_ = 3.10^e^, *p* > 0.05). Pairwise comparisons at each time point confirmed a significant increase in p-S831 at 20 min (Fig. 3*H*; *p* < 0.05) but not 2 h (Fig. 3*I*; *p* > 0.05). As with GluA2, p-S880 expression was not affected by stimulation at either time point (*p* > 0.05). We also found no significant effect of time or stimulation on phosphorylated serine 845, the PKA binding site for GluA1 (*p* > 0.05, data not shown). The phospho-assay results confirm a selective and time-dependent increase in GluA1 p-S831, a known gain-of-function modification of GluA1 conferred by PKC and CaMKII (Roche et al., 1996; Lee et al., 2000). Together with the subcellular localization data, the results suggests that nociceptive stimulation increases neural excitability in two ways: (1) by increasing GluA2-lacking localization to synaptic sites, and (2) by increasing the function of these inserted AMPARs through PKC/CamKII-phosphorylation (Ferguson et al., 2006; Hook et al. 2008).

### Nociceptive stimulation below SCI does not induce acute cell death in lumbar ventral horns

GluA2-lacking AMPARs have previously been shown to contribute to cell death of large motor neurons in the SCI lesion penumbra, leading us to ask whether INS modulates cell death in an experience-dependent fashion. We ran a panel of biomarkers for proteins associated with increased cellular activity, excitotoxicity, and/or apoptotic cell death (Fig. 3). Cytosolic subcellular fractions of ventral lumbar spinal homogenates were assessed for cFos, cJun, calpain I, and cleaved caspase 3 at 20 min, 2 h, or 24 h after stimulation. Despite a trend toward an early increase in cFos and cJun (Fig. 3*A*), and a later increase in calpain I and caspase 3 (Fig. 3*B*), ANCOVA (with beta-actin as covariate) revealed no significant effect of stimulation on any of these biomarkers (*F*_(1,65)_ < 0.53^f^, *p >* 0.05). These findings confirm that the synaptic effects observed in synaptoneurosomes were not likely from a small sample of cells that had survived a stimulation-induced cell death event. These findings also raise the possibility that the maladaptive effects of nociceptive input may be reversible.

### Nociceptive stimulation below SCI increases synaptic GluA1 and decreases synaptic GluA2 in the ventral horn neuropil in a time-dependent manner

**Figure 4.**
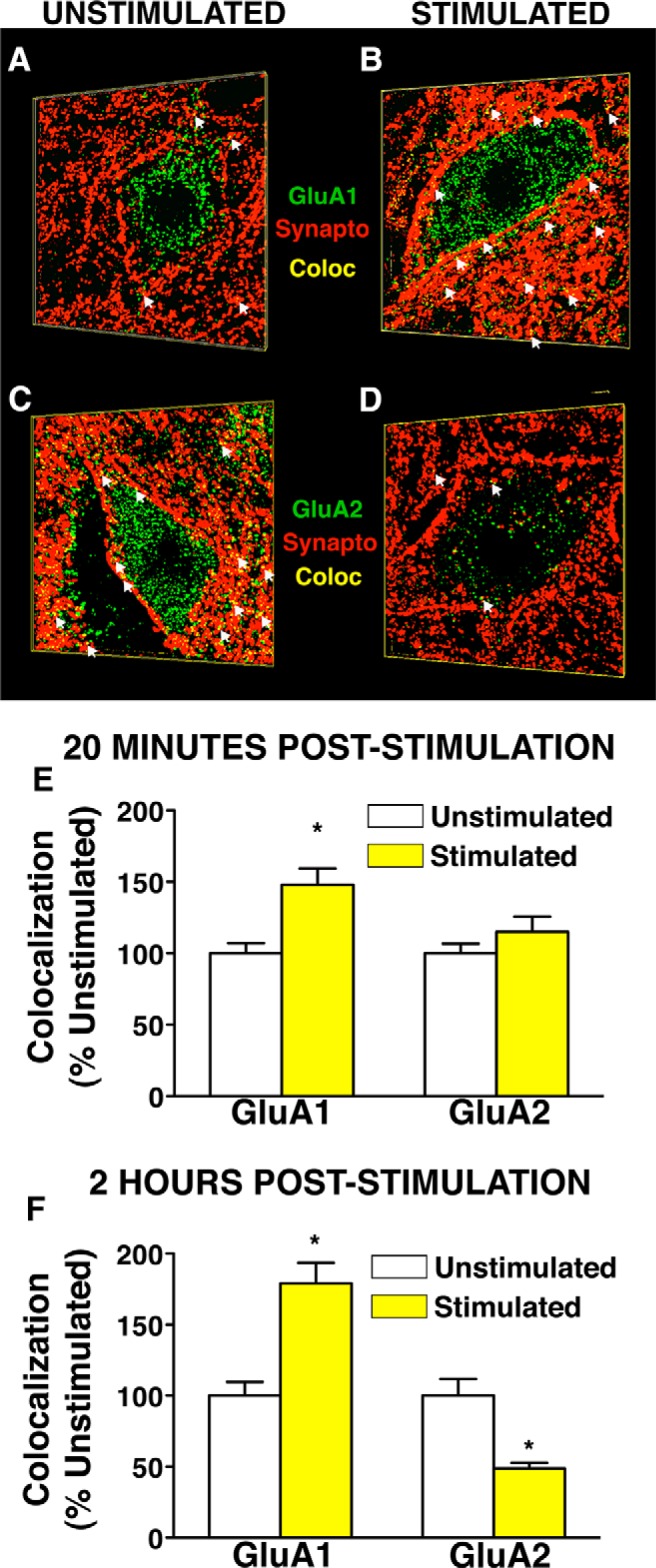
Synaptic GluA1 and GluA2 expression in 3-D synaptic field surrounding ventral horn neurons after intermittent nociceptive stimulation below complete spinal cord injury. Large ventral horn neurons in the L4–L5 region were assessed for colocalization of GluA1/2 (green) to synaptophysin-positive synapses (red) in nociceptive-stimulated and unstimulated spinally transected animals. ***A***–***D***, Representative 3-D confocal images of ventral horn neurons 2 h after stimulation or control demonstrating an increase in GluA1 expression (***A***, ***B***, green), and decreased GluA2 expression (***C*, *D***, green) after stimulation. Scale bar, 30 μm. ***E***, Quantification of GluA1 and GluA2 through confocal stacks at 20 min poststimulation shows a significant increase in synaptic GluA1 (*p < 0.05) and no change in GluA2. ***F***, Quantification of GluA1 and GluA2 through confocal stacks at 2 h poststimulation shows a significant increase in synaptic GluA1 and a concomitant decrease in synaptic GluA2 (*p < 0.05). Bars represent mean colocalization through confocal *z*-series of ventral horn neurons (124–146 cells per group assessed for GluA1, 105–154 cells per group assessed for GluA2; *n* = 4 subjects/per group; *N* = 16 rats total). Error bars represent standard error of the mean.

The observed synaptoneuromal changes in GluA1/GluA2 suggest a broad shift in total synaptic content toward a GluA2-lacking AMPAR profile; however, biochemical methods do not provide resolution at a single-cell level to assess whether these changes occur within a uniform population of cells to impact excitability. To test for GluA1/GluA2 ratio shifts in a well-phenotyped neuronal population of clinical significance, we targeted large motor neurons in the ventral horn. This was accomplished using a blinded, randomized analytical workflow of automated robotic high-resolution spinning disk confocal microscopy, followed by 3D blind iterative deconvolution, and automated image analysis to explicitly quantify synaptic and extrasynaptic AMPAR puncta on large ventral horn neurons and surrounding dendritic fields (Fig. 1). Large-scale analysis of >90,000 confocal images of large ventral motor neurons revealed a significant time-dependent increase in GluA1 puncta colocalized to synaptophysin-labeled synapses in INS-treated subjects relative to unstimulated controls (Fig. 4*A*,*B*). ANOVA confirmed main effects of stimulation and time for synaptic GluA1 (*F*_(1,536)_ > 30.20^g^, *p* < 0.01 and a significant Time × Stimulation effect for synaptic GluA2 (*F*_(1,493)_ = 18.62^h^; *p* < 0.01). Pairwise comparisons revealed that INS significantly increased synaptic GluA1 by 20 min poststimulation (*p* < 0.05), whereas GluA2 levels remained unchanged (Fig. 4*E*, *p* > 0.05). At 2 h poststimulation, synaptic GluA1 expression remained elevated, whereas synaptic GluA2 was significantly diminished (Fig. 4*F*; *p* < 0.05). The decrease in GluA2 between 20 min and 2 h after nociceptive stimulation suggests turnover of AMPAR subtypes from a calcium-impermeable GluA2-containing population to calcium-permeable GluA2-lacking AMPARs.

### Nociceptive stimulation decreases GluA2 expression first at extrasynaptic sites, then at synaptic sites on ventral horn neurons *in vivo*

**Figure 5.**
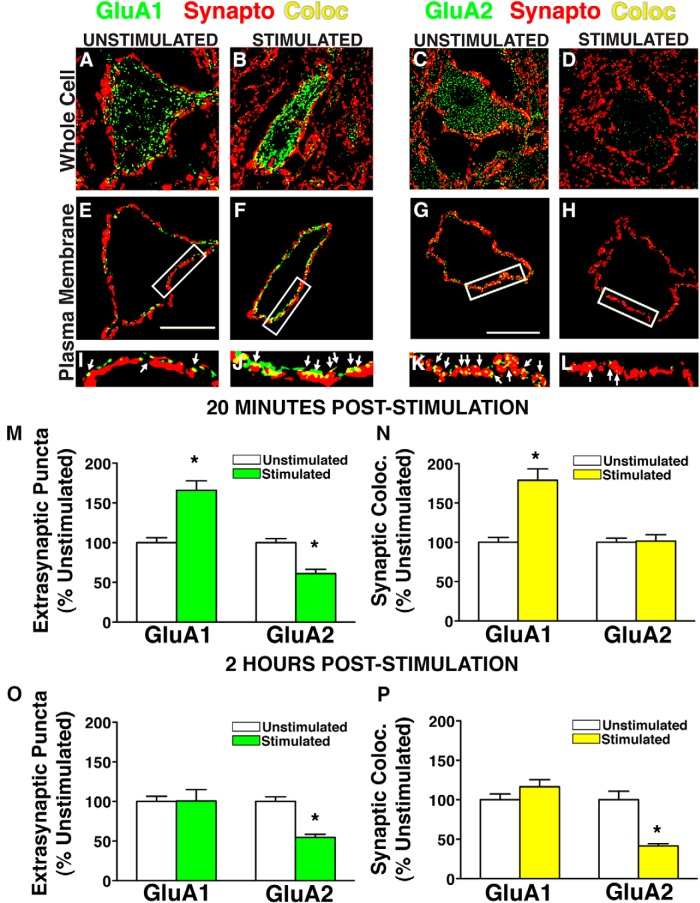
Ext`n of GluA1 and GluA2 on plasma membrane of ventral horn neurons after intermittent nociceptive stimulation below complete spinal cord injury. Large L4–L5 ventral horn neurons in were assessed for colocalization (yellow) of GluA1/2 (green) to synaptophysin-positive synapses (red) after nociceptive stimulation. Algorithmically selected single confocal planes of peak GluA1/synaptophysin colocalization for unstimulated (***A***) and stimulated groups (***B***), and GluA2/synaptophysin colocalization for unstimulated controls (***C***) and stimulated groups (***D***). ***E***–***H***, A 2-μm-wide cutout of the confocal image containing somatic plasma membrane. ***I***–***L***, Boxed plasma membrane fractions enlarged to illustrate representative differences in extrasynaptic (green) and synaptic (yellow) GluA1/2 puncta on motor neuron plasma membranes. ***M***–***P***, Quantification of extrasynaptic GluA1/2 puncta and synaptic colocalization of subunit puncta with synaptophysin. ***M***, Extrasynaptic GluA1 was significantly increased 20 min after stimulation, whereas extrasynaptic GluA2 is significantly decreased (ANOVA, *p < 0.05). ***N***, Synaptic colocalization of GluA1 and synaptophysin was also significantly increased (*p < 0.05), whereas synaptic GluA2/synaptophysin colocalization is unaltered by stimulation. ***O***, Two hours after stimulation, extrasynaptic GluA1 expression is unchanged between stimulated and unstimulated groups, while extrasynaptic GluA2 remains significantly decreased in response to stimulation (*p < 0.05). ***P***, Synaptic GluA1/synaptophysin colocalization is unchanged at 2 h poststimulation, but synaptic GluA2/synaptophysin colocalization is significantly decreased in response to stimulation (*p < 0.05). Bars represent means for 124–146 cells/group for GluA1, 105–154 cells per group for GluA2; *n* = 4 subjects/per group, *N* = 16 rats total. Error bars represent standard error of the mean.

**Figure 6.**
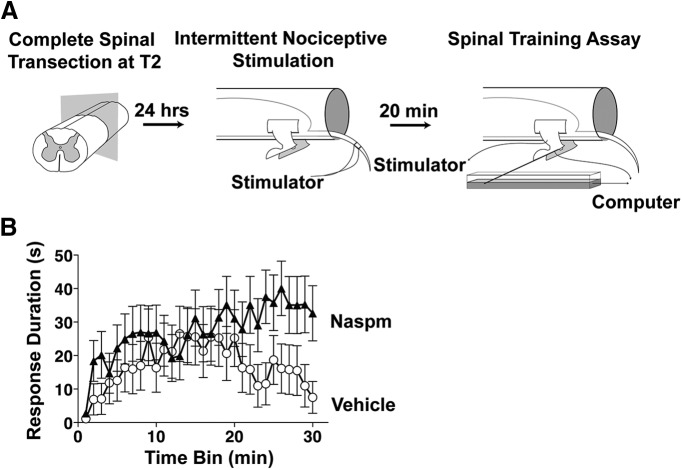
Effect of CP-AMPAR antagonist on impaired adaptive sensorimotor performance following intermittent nociceptive stimulation. ***A***, INS/spinal cord training paradigm. Rats with complete thoracic spinal transection received 6 min of INS to the tail followed by intrathecal administration of the CP-AMPAR antagonist Naspm (10 mm). Spinal instrumental training task began 20 min later. ***B***, Vehicle-treated subjects failed to exhibit a progressive increase in response duration over time, indicative of the INS-induced impairment in spinal adaptation. Naspm increased response duration over time, indicating that blocking CP-AMPAR activity protects against INS-induced maladaptive spinal plasticity. ANOVA revealed a significant increase in response duration over time in the Naspm-treated group compared to vehicle-treated animals, *n* = 12 subjects/per group (repeated measures, *p* < 0.05). Error bars represent standard error of the mean.

Cell culture work has demonstrated activity-dependent AMPARs trafficking occurs in distinct phases. First, AMPARs are trafficked from intracellular endosomes into extrasynaptic plasma membrane. They are then moved laterally into the synapse by means of a distinct molecular mechanism (Borgdorff and Choquet, 2002; Park et al. 2004). We sought to determine whether *in vivo* INS engages distinct time-dependent changes in extrasynaptic and synaptic distribution of GluA1 and GluA2 in the injured spinal cord. Using custom designed automated image analysis macros, we narrowed the focus of analysis to the plasma membrane compartment of lumbar ventral spinal motor neurons to assess the localization of GluA1 or GluA2 at extrasynaptic and synaptic sites (Fig. 5*A*–*L*). Automated analysis of a single optical plane (algorithmically-selected) from >2400 motor neurons revealed changes in GluA1 that were tightly coupled between extrasynaptic and synaptic sites, whereas GluA2 showed distinct temporal features at extrasynaptic and synaptic sites, resulting in a net increase in GluA1–GluA2 ratio (Fig. 5*A*–*L*), but through distinct mechanisms at different time points. At extrasynaptic sites, GluA1 rapidly increased and GluA2 decreased by 20 min post-INS (Fig. 5*M*). However, at synaptic sites only GluA1 increases were seen, with no change in GluA2 (Fig. 5*N*). By 2 h poststimulation persistent GluA2 removal was observed at both extrasynaptic and synaptic sites, whereas GluA1 changes returned to control levels (Fig. 5*O*,*P*). An ANOVA confirmed a significant Time × Stimulation interaction on GluA1 expression at extrasynaptic sites (*F*_(1,535)_ = 8.94^i^, *p* < 0.01) and main effects of time and stimulation at synaptic sites (*F*_(1,534)_ > 7.88^j^; *p* < 0.01). Pairwise comparisons revealed that stimulation significantly increased extrasynaptic and synaptic GluA1 at 20 min (*p* < 0.05), and that these effects waned by 2 h (*p* > 0.05). Analysis of GluA2 revealed significant main effects of time and condition at extrasynaptic sites (*F*_(1,493)_ > 15.16^k^, *p* < 0.01), and a significant Time × Stimulation interaction on synaptic GluA2 (*F*_(1,493)_ = 17.93^l^, *p* < 0.01). Pairwise comparisons revealed that INS significantly decreased extrasynaptic GluA2 20 min after stimulation (Fig. 6*M*; *p* < 0.01), followed by a similar decrease in both extrasynaptic and synaptic GluA2 expression by 2 h after stimulation (Fig. 5*O*,*P*; *p* < 0.01). These findings suggest that INS-induced maladaptive plasticity in the injured spinal cord may reflect an increased membrane expression of GluA2 lacking-AMPARs that is initiated extrasynaptically, and then expressed at synaptic sites by 2 h following stimulation. This has potential implications for timing of therapeutic interventions to reduce motor neuron hyperexcitability after SCI.

### Specific CP-AMPAR antagonist restores spinal motor training capacity in the face of nociceptive stimulation below a complete SCI

To assess behavioral consequences and therapeutic targeting of CP-AMPAR activity in INS-induced spinal plasticity we harnessed a behavioral assay of adaptive sensorimotor modification in the spinal cord (Grau et al., 1998, 2006). INS is known to impair spinal cord sensorimotor training and recovery of locomotor function after SCI (Crown et al., 2002a; Grau et al., 2004). To test whether CP-AMPAR over-activity mediates the INS-induced deficits in motor function, we intrathecally delivered a specific CP-AMPAR antagonist (Naspm) following INS and then tested spinal cord sensorimotor training capacity using a spinal instrumental (response-outcome) learning task. The spinal training assay requires rats with complete T2 spinal cord transection to increase the duration of hind-limb flexion (ie, the response) to reduce exposure to nociceptive electric shock (outcome; Fig. 6*A*). Given that the lumbar spinal cord is surgically isolated from the brain, this sensorimotor training occurs in the spinal circuitry itself providing an assay of endogenous spinal cord plasticity (Buerger and Fennessy, 1970; Grau et al., 1998).

Our findings indicate that INS induces an acute modification of AMPAR function, producing an increase in Ca^++^ permeability that we predict disrupts spinal sensorimotor learning. To explore this possibility, we assessed the impact of blocking CP-AMPARs using Naspm. NS administration impaired spinal learning in vehicle-treated subjects (Fig. 6*B*). Intrathecal delivery of Naspm immediately following INS produced a significant restoration of response duration over time (ANOVA, *F*_(1,29)_ = 1.64^m^, *p* < 0.05; Fig. 6*C*). This finding suggests that INS acutely undermines adaptive spinal plasticity through CP-AMPAR activity, and suggests that suppression of CP-AMPAR overdrive in the injured spinal cord may restore adaptive spinal synaptic modifications. Naspm has also been shown to be therapeutically effective when given 24 h after INS (Huie et al., 2012a).

## Discussion

Our results demonstrate that peripheral nociceptive input below an acute complete SCI induces a form of AMPA-mediated maladaptive synaptic plasticity that impairs future spinal cord training. Nociceptive stimulation directed GluA1 into ventral horn synaptoneurosomes and enhanced its PKC/CamKII site phosphorylation (p-S831). Confocal imaging revealed time-dependent increases in GluA1 and decreased GluA2 on large spinal motor neurons, with GluA2 removal occurring first at extrasynaptic sites followed by synaptic removal 2 h later. These findings suggest that nociceptive stimulation persistently increases trafficking of GluA2-lacking (calcium-permeable) AMPARs to spinal synapses. The specific CP-AMPAR antagonist Naspm protected against INS-induced failure in a spinal sensorimotor learning task, indicating the necessity of CP-AMPAR activity for maladaptive spinal plasticity. Together, these findings reveal synaptic GluA2-lacking AMPARs as mechanistic targets for restoring effective synaptic and behavioral plasticity in the injured spinal cord *in vivo*.

Prior *in vitro* work has revealed that activity-dependent CP-AMPAR expression is regulated by rapid receptor trafficking to synaptic sites in two stages: AMPARs first insert at extrasynaptic sites and then traffic laterally into the postsynaptic density (Ehlers, 2000; Bredt and Nicoll, 2003). Our findings indicate that nociceptive stimulation *in vivo* engages a similar trafficking cycle for CP-AMPARs in the injured spinal cord (Fig. 5 *M*,*P*). Data from models of neuronal insult suggest that injury-induced activation of PKC can mobilize the AMPA-associated protein PICK1 to the postsynaptic density, where it binds specifically to GluA2 subunits and promotes the internalization of GluA2-containing AMPARs (Liu et al., 2006). Thus, GluA2-lacking CP-AMPAR expression at postsynaptic sites is amplified though calcium-sensitive PKC activation, and may be sustained. Our finding that nociceptive stimulation increases PKC/CaMKII site phosphorylation suggests a phenotypic switch to sustained CP-AMPAR expression that is in line with prior observations that PKC inhibitors can restore adaptive spinal cord plasticity in the face of nociceptive stimulation (Malmberg et al., 1997; Zeitz et al., 2001; Ferguson et al., 2008a; Asiedu et al., 2011; King et al., 2012).

The current study builds on previous findings that both nociceptive stimuli and spinal cord injury saturate glutamatergic neurotransmission, undermining adaptive spinal motor plasticity (Tillakaratne et al., 2002; Chau et al., 2002; Ferguson et al., 2012b; Hoy et al., 2013). Prior work has also shown that both nociception and SCI generate inflammatory cytokines that amplify GluA2-lacking AMPAR tone (Ferguson et al., 2008b; Choi et al., 2010). INS below SCI has recently been shown to increase expression of the inflammatory cytokine tumor necrosis factor alpha (TNFα) leading to long-term impairment in spinal learning (Huie et al., 2012a). Prior *in vitro* studies revealed that TNFα drives AMPAR currents, specifically by increasing synaptic GluA2-lacking CP-AMPAR expression (Beattie et al., 2002; Stellwagen et al., 2005; Leonoudakis et al., 2008). Further, TNFα also increases CP-AMPAR expression *in vivo* to induce rapid motor neuron death near the site of SCI (Ferguson et al., 2008b). Inhibiting either TNFα or CP-AMPAR activity (using Naspm) 24 h after INS restores the capacity for adaptive sensorimotor lumbar learning (Huie et al., 2012a), suggesting that CP-AMPAR activity not only mediates the acute impact of nociceptive stimulation but also plays a key role in sustaining maladaptive plasticity over time. Naspm efficacy implies that INS does not impair lumbar-sacral learning simply because of enhanced spinal cord cell death through over-excitation. We tested this possibility by assessing a canonical apoptotic cell death marker (caspase 3), and a marker for calcium-dependent cell death (calpain I). We complemented these markers with cFos and cJun, two indicators of acute cell activity that have been shown in cell death models to be closely associated with both glutamatergic excitotoxicity and apoptosis (Anderson et al., 1995; Hermann et al., 2001; Xu et al., 2005). Western blot analysis in the present paper showed that INS did not significantly increase expression of cFos, cJun, calpain I, or caspase 3. The lack of biochemical evidence for INS inducing cell death distal to injury indicates that INS drives a therapeutically reversible form of maladaptive spinal cord plasticity in the lumbar circuitry controlling movement. In contrast, work focusing on the lesion penumbra has found that INS enhances development of ongoing apoptosis and cell loss (Grau et al., 2004; Garraway et al., 2014). Although the impact of CP-AMPARs in this effect remains unclear, it is possible that Naspm could reduce excitotoxic components of secondary injury in addition to its impact on lumbar sensorimotor training, yielding dual therapeutic effects.

**Figure 7.**
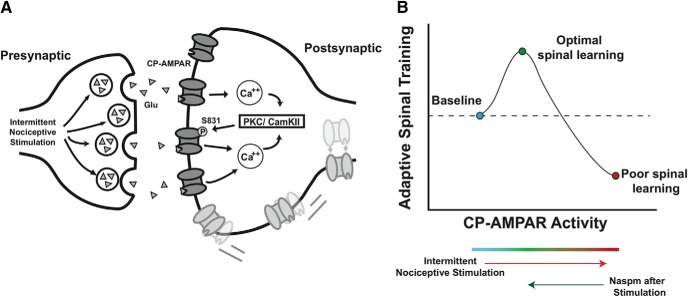
***A***, Theoretical pathway underlying INS-induced maladaptive plasticity. Following afferent intermittent nociceptive stimulation, increased glutamate release engages postsynaptic AMPA receptors. Calcium influx via CP-AMPARs activates the calcium detectors PKC and/or CamKI phosphorylating the serine 831 site on GluA1 AMPAR subunit. Serine 831 phosphorylation increases the open probability of AMPARs, creating a feedforward loop that leads to membrane insertion of extrasynaptic CP-AMPARs. These receptors are trafficked laterally to the synaptic membrane, further strengthening this excitatory connection. ***B***, Conceptual model of CP-AMPAR effects on spinal plasticity after SCI. CP-AMPAR activity critically shapes synaptic strength and use-dependent spinal cord plasticity after injury. Peripheral stimulation below the injury engages CP-AMPAR-mediated calcium influx, activating intracellular modulators of synaptic plasticity and strengthening excitatory tone to promote adaptive spinal training. However, CP-AMPARs are hyper-responsive to peripheral input (eg, limb positioning; skin stimulation) and are easily overdriven, resulting in synaptic saturation that overwhelms the capacity for adaptive spinal cord learning. As CP-AMPAR activity further increased, excitotoxicity and cell death may occur. Therapeutic intervention to decrease CP-AMPAR over-activity normalizes the balance of synaptic GluA1 and GluA2, and restores optimal adaptive plasticity.

Based on theoretical and experimental evidence, we suggest a working model that INS alone first engages GluA2-lacking AMPARs resulting in calcium influx and activation of calcium detectors PKC and CamKII (Roche et al. 1996; Barria et al., 1997; Gómez-Pinilla et al., 2007; Ferguson et al., 2008a; Fig. 7*A*), which phosphorylate S831 to further strengthen synaptic GluA1 drive, but not GluA2 (Fig. 2). For vehicle-treated subjects, the training session following INS exposure further strengthens this maladaptive plasticity. In contrast, Naspm treatment resets the metaplastic state of the spinal cord, giving subjects the opportunity to exhibit an adaptive spinal learning response, initiating a second wave of AMPAR-mediated plasticity that involves a greater number of GluA2-containing AMPARs. The data suggest that INS followed by a combined therapeutic effect of Naspm and spinal cord training, re-engages the PKC pathway, as evidenced by additional increases in both pS831 and GluA1 expression. Together, the data suggest that Naspm and adaptive training produce an optimal balance of AMPAR drive for spinal cord learning (Fig. 7*B*).

This model is consistent with the idea that both CP-AMPAR over-activity and underactivity may be problematic. Naspm treatment in cultured hippocampal neurons may block the normal rebound of synaptic strength following tetrodotoxin administration, indicating that CP-AMPARs are necessary for homeostatic plasticity in the brain (Hou et al., 2011). CP-AMPAR activity may produce a continuum of synaptic and behavioral effects, wherein moderate AMPAR-mediated calcium influx can be beneficial and necessary for adaptive plasticity (as in the stabilization of LTP in the brain, and in performance on the adaptive spinal sensorimotor task), but as CP-AMPAR activity increases in response to insult or nociceptive input, an excitatory saturation level is reached at which point spinal neurons lose the capacity to encode subtle simulation patterns, as in the adaptive spinal training task (Fig. 7*B*). Therapeutic interventions to restore homeostatic balance following injury or insult will likely be key in promoting the optimal glutamatergic regulation of adaptive plasticity. A growing body of evidence has shown that AMPAR antagonists including Naspm restore neural function and suppress excitotoxic cell death in models of epilepsy, ischemia, neuropathic pain, opioid-induced hypersensitivity, and motor neuron diseases (Sorkin et al., 1999, 2001; Van Damme et al., 2003; Walters et al., 2005; Leonoudakis et al., 2008; Spaethling et al., 2008; Yin et al., 2012; Cabanero et al., 2013; Dias et al., 2013).

The current study contributes to these findings by presenting a possible cellular mechanism by which peripheral nociceptive input drives maladaptive spinal cord plasticity. The high incidence of polytrauma associated with SCI in the human population (Saboe et al., 1991; Sehkon and Fehlings, 2001; Wang et al., 2001; Hasler et al., 2011) highlights the need to understand the impact of nociceptive input in the injured spinal cord. Beyond clearly-defined peripheral injuries, growing evidence suggests that the injured spinal cord may also be sensitive to aberrant peripheral input, including limb immobilization and stretching (Caudle et al., 2011, 2015). Recent work in SCI patients demonstrates that c-fiber activation impacts retention of motor learning tasks (Bouffard et al., 2014). The present results provide a mechanism for these clinical findings, suggesting that CP-AMPARs represent a therapeutic target. The data presented in this study provide insight into maladaptive spinal plasticity in an isolated spinal system at acute time points following a complete SCI. Future work on CP-AMPARs in other SCI models and at chronic time points will be invaluable for testing the therapeutic potential of targeted CP-AMPAR antagonism for mitigating maladaptive plasticity to promote functional recovery after spinal cord injury.

## Synthesis

The decision was a result of the Reviewing Editor Michael Sofroniew and the peer reviewers coming together and discussing their recommendations until a consensus was reached. A fact-based synthesis statement explaining their decision and outlining what is needed to prepare a revision is listed below. The following reviewers agreed to reveal their identity: David Magnuson

Reviewer #1:

In this manuscript the authors evaluate the expression and role of AMPA receptor subunits following a complete SCI and application of nociceptive stimulation which results in maladaptive plasticity within spinal motor systems.

Peripheral nociception can negatively alter both spinal dorsal horn and motor neurons. This can result in impaired or undermined recovery. The main goal of this manuscript is to investigate a potential mechanism behind this phenomenon. AMPA receptor plasticity after injury is one such possible factor. Given the dynamic regulation after injury, the authors hypothesize that nociceptive input overly enhances calcium permeable (GLuR2 lacking) AMPA-R's resulting in an overwhelmed system. To test this hypothesis the authors utilized quantitative biochemical and imaging techniques to evaluate CP-AMPA-Rs levels and synaptic localization after injury and application of an INS protocol. Indeed the authors find that CP-AMPA-Rs are increased. However, when CP-AMPA-Rs were blocked proper adaptive responses were restored.

Overall, this is a very interesting topic focused on processes which can significantly impact recovery after SCI. Understanding these processes will allow for future refinement of potential interventions. The authors should also be commended for their rigorous approaches used in this study.

This is a well written manuscript with only minor points to consider:

1) The authors should consider using another word than "drug-able" which is used in both the Abstract and Introduction.

2) The authors include to total N, but do not include n's for each sub-experiment/group. Please include this.

3) Did the authors provide analgesics to the animals post injury? This is not stated. If so, is there a potential for analgesics to impact the results? Also it reads in the Spinal Transection Surgery section as if the animals were maintained post injury for multiple days, but were kept for only 24 hours post SCI. Please fix.

4) The authors should describe the stimulation delivery more clearly (i.e. each pulse was delivered every 2 s for 80 ms - or something like that). Also, it is not clear what was the control for INS. It is clear from reading later in the manuscript how this was controlled for, but this should be included in the Materials and Methods section.

5) The authors should also include when the spinal sensorimotor task was performed. Again this was noted elsewhere but not in the Materials and Methods section.

6) The authors include a very thorough Discussion section but the authors should be careful in how they frame their study. As the current study is in the acute stages of injury, it is not clear what the impact is in chronic SCI. Questions to ask in future studies include: how persistent are these maladaptive plastic changes or alternatively, how long after injury can one produce maladaptic plastic responses?

Reviewer #2:

This is a very well written manuscript that describes a series of studies focused on maladaptive plasticity after a spinal cord injury and the potential role that nociceptive input plays in driving that maladaptive plasticity. In addition, the manuscript presents a potential mechanism behind the changes, in particular at the level of the motoneurons and the synapses they receive, and a model by which other mechanisms and pharmacological therapeutics might be tested. This work obviously has great potential significance for the management of those with spinal cord injuries because it provides additional solid evidence that nociceptive input, below the level of injury, may have a dramatic impact on adaptive plasticity and learning (ie. recovery) after injury.

The study design itself is outstanding, with the caveats discussed below, and the results provide a convincing story that INS (intermittent nociceptive stimulation), as a model of nociceptive input, leads to substantial changes in AMPA receptor subunit composition and density and that these changes happen in the absence of changes in markers for cell activation (fos and jun) or cell death (caspase and calpain). These aspects of the study and the manuscript are very solid and allow the authors to put forward a convincing model, that they, in-turn, test pharmacologically using Naspm, a soluble AMPAR antagonist, showing that it protects the spinal circuitry against intermittent nociceptive stimulation-induced maladaptive plasticity.

Caveats: The primary concern with this manuscript is, in fact more of a concern with the primary model being studied, being a fully transected rat over the initial 24-48 hours post-transection. While this model has proved to be very powerful and amenable to provide information about plasticity and nociceptive influence on spinal circuitry acutely post-injury, the study is limited to this early acute (hyper-acute?) period, which may not be indicative of how the system will respond at later, and likely more relevant, time points. This primary caveat should be included and some of the statements regarding the key results should be worded more carefully to clearly indicate the time frame of the changes.

Minor issues:

1.Related to above, the initial statement in the discussion section states that the investigators have identified a "lasting" form of AMPA receptor related plasticity. While this may be true, the term "lasting" requires some qualification to put the work in the correct context. This doesn't decrease the potential impact of the findings.

2.Also in the early part of the discussion, referring to c-fos and c-jun simply as cell death markers, and lumping them together with calpain and caspase 3 is over-simplistic. This data is quite germaine to the study and is worth a little more effort to provide appropriate context. C-fos and jun have long been thought of as activity-indicators, so please provide more context, including references, for this data.
